# Combining a Disturbance Observer with Triple-Loop Control Based on MEMS Accelerometers for Line-of-Sight Stabilization

**DOI:** 10.3390/s17112648

**Published:** 2017-11-17

**Authors:** Yong Luo, Yongmei Huang, Chao Deng, Yao Mao, Wei Ren, Qiongyan Wu

**Affiliations:** 1 Key Laboratory of Optical Engineering, Chinese Academy of Sciences, Chengdu 610209, China; ly250047087@126.com (Y.L.); huangym@ioe.ac.cn (Y.H.); chaosir1991@gmail.com (C.D.); renwei9327@163.com (W.R.); wuqiongyan@21cn.com (Q.W.); 2 Institute of Optics and Electronics, Chinese Academy of Science, Chengdu 610209, China; 3 University of Chinese Academy of Sciences, Beijing 100039, China

**Keywords:** MEMS accelerometers, triple-loop control, composite velocity loop, disturbance observer, line-of-sight, disturbance suppression ability

## Abstract

In the CCD-based fine tracking optical system (FTOS), the whole disturbance suppression ability (DSA) is the product of the inner loop and outer position loop. Traditionally, high sampling fiber-optic gyroscopes (FOGs) are added to the platform to stabilize the line-of-sight (LOS). However, because of the FOGs’ high cost and relatively big volume relative to the back narrow space of small rotating mirrors, we attempt in this work to utilize a cheaper and smaller micro-electro-mechanical system (MEMS) accelerometer to build the inner loop, replacing the FOG. Unfortunately, since accelerometers are susceptible to the low-frequency noise, according to the classical way of using accelerometers, the crucial low-frequency DSA of the system is insufficient. To solve this problem, in this paper, we propose an approach based on MEMS accelerometers combining disturbance observer (DOB) with triple-loop control (TLC) in which the composite velocity loop is built by acceleration integration and corrected by CCD. The DOB is firstly used to reform the platform, greatly improving the medium-frequency DSA. Then the composite velocity loop exchanges a part of medium-frequency performance for the low-frequency DSA. A detailed analysis and experiments verify the proposed method has a better DSA than the traditional way and could totally substitute FOG in the LOS stabilization.

## 1. Introduction

CCD-based fine tracking optical systema (FTOS) are being applied more and more to point and stabilize the line-of-sight (LOS) in many applications, such as adaptive optics, laser communication, astronomical observation and quantum communication [1,2,3,4,5]. Generally, the tracking bandwidth and the disturbance suppression ability (DSA) are two key indicators of the FTOS. However, due to the low CCD sampling rate, time delay of image processing and the mechanical resonance of the platform, it is nearly impossible to acquire a wide bandwidth [6,7], so many scholars have focused on approaches to increase the DSA of the system. As the whole DSA is the product of the inner loop and outer position loop [8], Traditionally, the high sampling fiber-optic gyroscope (FOG) are added to the platform to build the velocity dual-loop control (VDLC) including position and velocity loops for the LOS stabilization [9,10,11]. However, high-performance FOGs are expensive and relatively large compared to the back narrow space of the small rotating mirror, and consume significant power to precisely control their fiber-coil temperatures, which limits their utilization.

With the development of the micro-electro-mechanical system (MEMS) industry, the performance of inertial sensors, such as MEMS accelerometers and gyroscopes with small size, low weight, low cost and low power consumption, especially accelerometers whose bandwidth could reach 1000 Hz, has improved a lot [12,13]. However, since the MEMS gyroscope’s bandwidth usually doesn’t exceed 100 Hz, so it is unable to substitute the FOG alone whose bandwith is about several hundred Hz in FTOS. Therefore, In 2016, Tian combined a MEMS gyroscope with a MEMS accelerometer to construct a triple-loop control (TLC) which includes acceleration, velocity and position loops to enhance the DSA and succeeded in making the whole DSA equal to the traditional way [8]. Nevertheless, in the space limited occasion, the rotating mirror is too small to install two different sensors, so we try to use only a sensor, obviously only the high-bandwidth MEMS accelerometer has the potential to substitute the FOG in the system.

Since Studenny and Belanger firstly introduced accelerometers to the feedback control system [14], more and more researchers began to use accelerometers in precision control field [12,15,16]. In 2009, Tang introduced the accelerometers to FTOS. As accelerometers are susceptible to the low-frequency noise, he designed the acceleration closed loop to be a band-pass filter to guarantee the system’s stability, which resulted in its insufficient DSA in low frequency compared with the traditional VDLC way [17]. However, the DSA in low frequency is crucial for the system, because in most occassions the low-frequency and medium-frequency disturbance is primary.

In this paper, in order to enhance the DSA with accelerometers, we propose an approach based on MEMS accelerometers combining the disturbance observer (DOB) built in the acceleration loop with TLC in which the composite velocity loop is built by acceleration integration and corrected by CCD. Since the low-frequency disturbance observed by DOB is not accurate because of accelerometer’s noise and the model of FTOS is not fully matched with the real platform at high-frequency, we use the DOB method mainly to greatly improve the medium-frequency DSA. Then, we try to add a composite velocity loop to improve the low-frequency DSA, whose difficulty is how to acquire the velocity without gyroscopes. Due to the accelerometers’ low-frequency noise, the pure integration of acceleration to get the velocity would result in the velocity’s drift. Accoarding to the proposed correction method, we can get a velocity without drift, whereas the CCD’s correction would bring time delay to the velocity’s loop, which is bad for the medium frequency DSA but have little effect on the low-frequency DSA. However, it’s not a problem, since the DOB method has much reformed the system, the whole DSA in medium frequency is still good and the essence is that we exchange the system’s a part of medium-frequency performance for the low-frequency DSA. At last, we can get a better DSA nearly in the whole frequency domain than the traditional way of FOG.

This paper is organized as follows: Section 2 presents a detailed introduction to CCD-based FTOS structure, the traditional control way and the basic implementation of the proposed method. Section 3 concentrates on the DOB controller design and its performance analysis. Section 4 introduces how to accomplish a composite velocity loop. Section 5 is the experiment part to verify this method effective. Concluding remarks are presented in Section 6.

## 2. CCD-Based Fine Tracking Optical System

**Figure 1:**
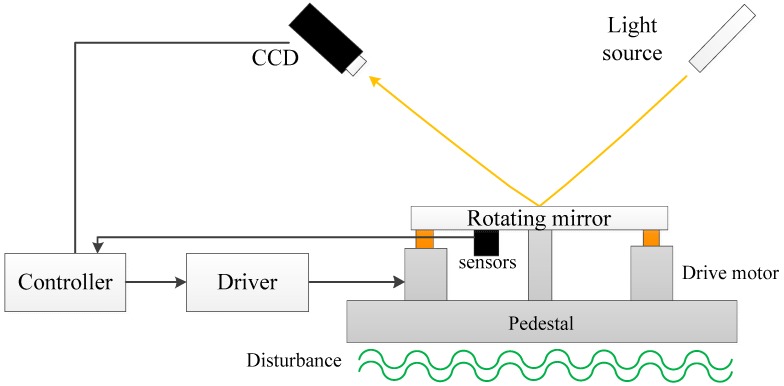
The configuration of the FTOS.

The configuration of the FTOS is illustrated in Figure 1. The light source emits light to simulate the target, which is detected by a CCD to calculate the LOS error. The inertial sensors like MEMS accelerometers and FOG could acquire the angular acceleration and velocity of the mirror. The controller receives the feedback signals, implements control algorithm and controls the motors to track the target and suppress the disturbance from the pedestal.

The FTOS acceleration open-loop transfer function as follows is composed of a quadratic differential element, a mechanical resonance part with natural frequency ${\tilde{\omega }}_{n}$, and a inertial element with electrical time constant *T_e_* [8,18]:

(1) $$\begin{array}{l} G_{a}(s)=\frac{\theta (s)}{U(s)}=\frac{Ks^{2}}{\frac{s^{2}}{{\tilde{\omega }}_{n}^{2}}\;+\;\frac{2\xi }{{\tilde{\omega }}_{n}}s\;+\;1}\cdot \frac{1}{T_{e}s\;+\;1} \end{array}$$

### 2.1. The Traditionnal Feedback Control Way in FTOS

**Figure 2:**
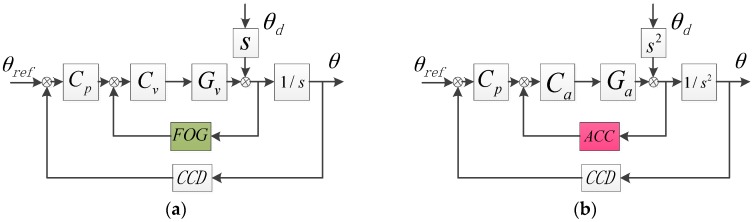
The feedback control structure. (**a**) VDLC; (**b**) ADLC. $G_{v}$ and $G_{a}$ are respectively the velocity and acceleration open-loop transfer function. $C_{v}$, $C_{a}$ and $C_{p}$ are velocity, acceleration and position controllers, $\theta_{ref}$ is the given position of target, $\theta_{d}$ is the outer disturbance and θ is the output.

Figure 2 respectively exhibits the traditional velocity dual-loop control (VDLC) and the pure acceleration dual-loop control (ADLC) raised by Tang in 2009 [17].

The two methods’ transfer function of DSA is as follows:

(2) $$E_{VDLC}=\frac{\theta }{\theta_{d}}=\frac{1}{1{\;+\;C}_{v}G_{v}}\cdot \frac{1}{1{\;+\;C}_{P}\frac{C_{v}G_{v}}{1{\;+\;C}_{v}G_{v}}\frac{1}{s}}\approx \frac{1}{1{\;+\;C}_{v}G_{v}}\cdot \frac{1}{1{\;+\;C}_{P}\frac{1}{s}}$$

(3) $$E_{ADLC}=\frac{\theta }{\theta_{d}}=\frac{1}{1{\;+\;C}_{a}G_{a}}\cdot \frac{1}{1{\;+\;C}_{P}\frac{C_{a}G_{a}}{1{\;+\;C}_{a}G_{a}}\frac{1}{s^{2}}}\approx \frac{1}{1{\;+\;C}_{a}G_{a}}\cdot \frac{1}{1{\;+\;C}_{P}\frac{1}{s^{2}}}$$

In Equations (2) and (3), if the controllers can be designed ideally, the DSA of the two methods will be equal. However, the controller is not ideally designed in practice due to the accelerometers’ noise. For example, Tang has designed the inner closed-loop to be a band-pass filter that lead to an insufficient DSA in low-frequency, so unquestionably, according to the only feedback way of using accelerometes, it can’t substitute the FOG’s role in the system.

### 2.2. The DOB Method Built in the Acceleration Loop

**Figure 3:**
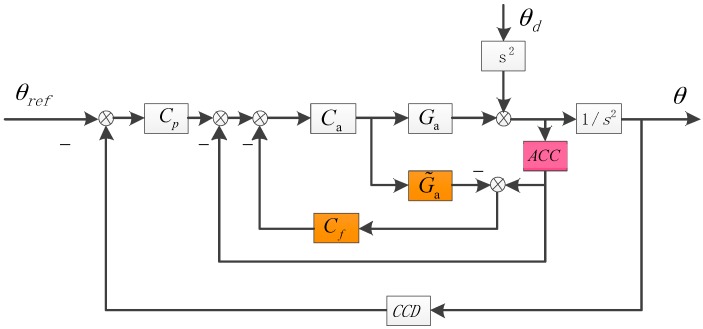
The structure of the ADLC with DOB. ${\tilde{G}}_{a}$ is the approximate acceleration model of the FTOS. $C_{f}$ is the DOB controller.

Nowadays, the DOB method has got more and more appalications in the industry control for it can estimate and compensate the external disturbance, which could apparently enhance the system’s robustness [19,20]. It’s very suitbale to the FTOS because we can precisely bulid the model by physical analysis and spectrum measurement [18,21]. The structure of the ADLC with DOB is presented as Figure 3.

Similarly, we can deduce the DSA in Figure 3 as follows:

(4) $$E_{ADLC-DOB}=\frac{\theta }{\theta_{d}}=\frac{1-C_{a}{\tilde{G}}_{a}C_{f}}{1+C_{a}G_{a}+C_{p}C_{a}G_{a}\frac{1}{s^{2}}+C_{a}(G_{a}-{\tilde{G}}_{a})C_{f}}\approx \frac{1}{1{\;+\;C}_{a}G_{a}}\cdot \frac{1-C_{a}{\tilde{G}}_{a}C_{f}}{1+C_{p}\frac{1}{s^{2}}}$$

Because ${\tilde{G}}_{a}$ is highly similar to the actual transfer function $G_{a}$, especially in low and medium frequency, the denominators of $E_{ADLC}$ and $E_{ADLC-DOB}$ are almost equal, which means the introduction of DOB has little effect on the stability of the system. If we set the $C_{f}$ to be the reverse of $C_{a}{\tilde{G}}_{a}$, the numerator of $E_{ADLC-DOB}$ will be 0 in theory, which signifies the influence of outer disturbance is almost eliminated by the DOB method. However, in fact, the improvement of the DSA by the DOB is restricted. At first, since in high-frequency domain the approximate acceleration mode ${\tilde{G}}_{a}$ is not very accurate, generally, we choose to compensate outer disturbance in low and medium frequency, abandoning the part of high frequency. Maybe this factor doesn’t have a lot influence on the DSA because the high-frequency DSA mainly relies on the passive DSA. But secondly, in the low frequency, the noise of accelerometers will dirty the observed disturbance signal, leading to not apparent improvement of DSA in low frequency. Therefore, the DOB method is mainly used to reform the medium-frequency performance and we need to look for a new way to further enhance the DSA in low frequency in order to make the MEMS accelerometers substitute the FOG.

### 2.3. The Composited Velocity Closed-Loop Built by MEMS Accelerometers and CCD

**Figure 4:**
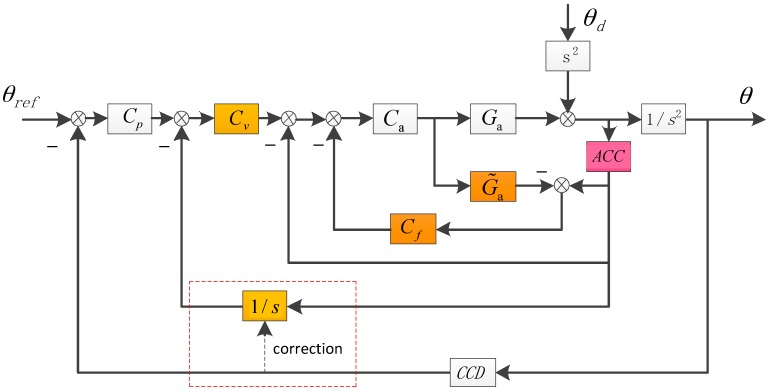
The structure of the TLC with DOB. The velocity is acquired by the acceleration integral and CCD signal would help to correct the velocity. $C_{v}$ is the velocity controller.

As we know, since the whole DSA is the product of each loop, it will continue to improve if we add a velocity loop to the structure. Fortunately, without gyroscopes, we can also acquire the velocity through the acceleration integral, but meanwhile the integral of the low-frequency acceleration noise will lead to drift of the velocity, which is harmful to the system’s stability. Although the common low-frequency cut-off or attenuation algorithm could eliminate the drift, it would also filter out useful signals, which seciously affects the system’s performance, so based on the acceleration integral, we propose to use the CCD signal to correct the velocity. The structure of TLC with DOB method is descripted in Figure 4.

The DSA of TLC with DOB is showed as follows:

(5) $$\begin{array}{ll} E_{TLC-DOB}=\frac{\theta }{\theta_{d}} & =\frac{1\;-\;C_{a}{\tilde{G}}_{a}C_{f}}{1\;+\;C_{a}G_{a}\;+\;C_{v}C_{a}G_{a}\frac{1}{s}\;+\;C_{p}C_{v}C_{a}G_{a}\frac{1}{s^{2}}\;+\;C_{a}(G_{a}\;-\;{\tilde{G}}_{a})C_{f}}\\ & \approx \frac{1}{1{\;+\;C}_{a}G_{a}}\cdot \frac{1}{1\;+\;C_{v}\frac{1}{s}}\cdot \frac{1\;-\;C_{a}{\tilde{G}}_{a}C_{f}}{1\;+\;C_{p}\frac{1}{s}} \end{array}$$

It’s easy to see that $E_{TLC-DOB}$ is smaller than $E_{ADLC-DOB}$, which means the added velocity loop would enhance the DSA of the system. However, due to the time delay of velocity loop brought by CCD’s correction, the actual improvement of DSA would only distribute at low frequency.

In FTOS, the CCD’s time delay is fixed and about 0.02 s (two frames whose sampling rate is 100 Hz). Take 1 Hz into consideration, the phase lag brought by time delay is (1/100)∗2π=π/50, which could be ignored. Although the bad effect on DSA by time delay becomes bigger as the frequency rises, but since the DOB has greatly improved the medium-frequency DSA and the high-frequency DSA is mainly determined by passive DSA relying on mechanical design, the last whole DSA is satisfied. Actually we choose to sacrifice a part of medium-frequency performance for the low-frequency DSA. The detailed procedures to get the composited velocity will be presented in Section 4.

## 3. The DOB Controller Design and Performance Analysis

Before designing the DOB controller, we first need to present the inner acceleration controller. In this paper, $C_{a}$ is designed as follows:

(6) $$C_{a}=\frac{\frac{s^{2}}{{\tilde{\omega }}_{n}^{2}}+\frac{2\xi }{{\tilde{\omega }}_{n}}s+1}{s}\cdot \frac{K_{a}}{T_{1}s+1}$$

The quadratic differential element is to compensate the paltform’s mechanical resonance, the integrator is used to partly compensate the quadratic differential of the acceleration object, and the inertial element is a low-pass filter to eliminate the high-frequency noise, in which $T_{1}$ should be very small. Thus, $C_{a}{\tilde{G}}_{a}$ is presented as Equation (7):

(7) $$C_{a}{\tilde{G}}_{a}=(\frac{\frac{s^{2}}{{\tilde{\omega }}_{n}^{2}}+\frac{2\xi }{{\tilde{\omega }}_{n}}s+1}{s}\frac{K_{a}}{T_{1}s+1})\cdot (\frac{Ks^{2}}{\frac{s^{2}}{{\tilde{\omega }}_{n}^{2}}+\frac{2\xi }{{\tilde{\omega }}_{n}}s+1}\frac{1}{T_{e}s+1})=\frac{KK_{a}s}{\left(T_{1}s+1)(T_{e}s+1\right)}$$

According to the analysis in Section 2.2, the ideal DOB controller is descripted as follows:

(8) $$C_{f}={\left(C_{a}{\tilde{G}}_{a}\right)}^{-1}=\frac{\left(T_{1}s+1)(T_{e}s+1\right)}{KK_{a}s}$$

In Equation (8), since the order of numerator is higher than the one of denominator, it cannot be accomplished in physics. Therefore, we need to transform the $C_{f}$ to be realizable:

(9) $$C_{f}=\frac{\left(T_{1}s+1)(T_{e}s+1\right)}{KK_{a}s}\approx \frac{T_{1}s+1}{KK_{a}s}\;(T_{e}<<1)$$

In Equation (9), as $T_{e}$ is much smaller than 1, the simplification is reasonable in low and medium frequency, resulting in sacrificing some DSA in high-frequency domain, which can be acceptable.

As the DSA improvement brought by DOB is owing to the numerator of $E_{TLC-DOB}$, we use $E_{DOB}$ to represent the numerator. Now we focus on the value of the numerator presented as follows:

(10) $$\begin{array}{ll} E_{DOB}=1-C_{a}{\tilde{G}}_{a}C_{f} & =1-\left(\frac{KK_{a}s}{\left(T_{1}s\;+\;1)(T_{e}s\;+\;1\right)}\right)\cdot \left(\frac{T_{1}s\;+\;1}{KK_{a}s}\right)\\ & =1-\frac{1}{T_{e}s\;+\;1}=\frac{T_{e}s}{T_{e}s+1} \end{array}$$

**Figure 5:**
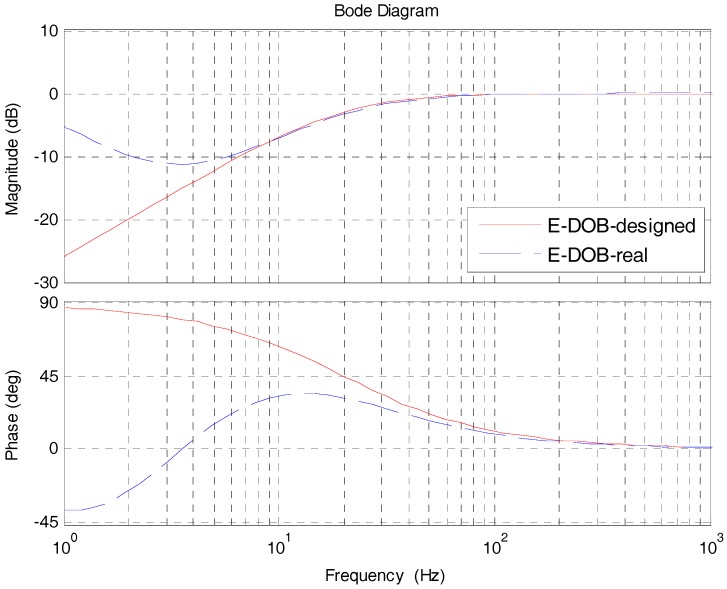
The simulation of $E_{DOB}$.

Obviously, $E_{DOB}$ is a high-pass filter. The red line in Figure 5 is the simulation of $E_{DOB}$. The improvement of DSA is apparent in low and medium frequency, and as the frequency goes down, the effect is stronger, while in high-frequency there is no improvement which meets our design. However, in fact, the actual $E_{DOB}$ will not exactly fit the red line. Since in low frequency the acceleration signal is weak which is susceptible by noise, the observed disturbance by accelerometers is not very accurate which leads to insufficient improvement of DSA in low frequency. Considering that, the blue line is the real $E_{DOB}$, which will rise as the frequency goes down. That is the reason we continue to add a composited velocity loop to enhance the DSA in low frequency.

## 4. The Accomplishment of the Composited Velocity Loop

We set the continuous function a(t) to represent the real acceleration of the platform. Generally, in low frequency, the noise is very big that we cannot ignore them which is descripted by ξ and during a sampling time we can treat ξ as a constant. The integral of the accelerometers’ signal is presented as follows:

(11) $$\tilde{V}(t)=\int \left(a(t)+\xi \right)dt=\int a(t)dt+\xi \cdot t=V(t)+\xi \cdot t$$

Obviously, $\tilde{V}(t)$ contains a drift except for the real velocity V(t). Thus, we decide to use the CCD’s position signal to correct it. However, at first, we need to acquire the estimated position by the integral of $\tilde{V}(t)$. The calculated position is as follows:

(12) $$\theta (t)=\int \tilde{V}(t)dt=\int \left(\int a(t)dt+\xi \cdot t\right)dt=\int V(t)dt+\frac{1}{2}\xi t^{2}$$

If we set S(t) to be the real position signal of CCD, from Equations (11) and (12), we can deduce the real velocity V(t):

(13) $$V(t)=\tilde{V}(t)+2\cdot \frac{S(t)-\theta (t)}{t}$$

**Figure 6:**
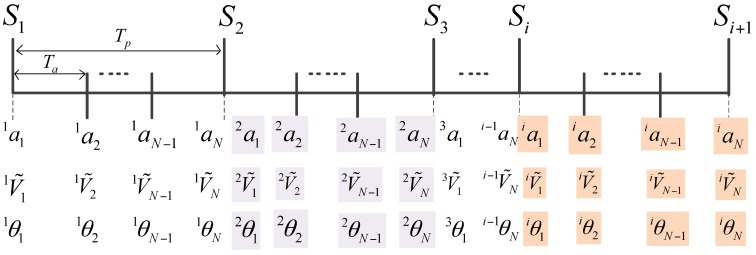
The sketch map of data update.

Since the computers cannot process the continuous signal, we transform these equations into discrete forms with trapezoidal discretization method. The sampling period of the inner acceleration loop and the estimation of position is $T_{a}$, which is much smaller than the CCD’s sampling period $T_{p}$. We assume $T_{p}=(N-1)T_{a}$, which means during a sampling period of the CCD, the accelerometers would produce N sampling data. Figure 6 exhibits the sketch map of data update.

The discrete forms of Equations (11) and (12) are as follows. The superscript represents the order of the CCD sampling period, and the subscript is the acceleration’s order:

(14) V˜ij=V˜ij−1+aij−1+aij2⋅Ta

(15) θij=θij−1+V˜ij−1+V˜ij2⋅Ta

Since there are drift in V˜ji and θji, when every CCD signal comes, we need to correct them. The recursive forms are as follows:

(16) {V˜i1=V˜i−1N+2⋅Si−θi−1NTpθi1=Si

In the first sampling period, we can set the V˜1i and θ11 to be 0, and with the above 4 recursive equations, we will get the composite velocity. As the inner acceleration loop has improved platform characteristics, the traditional PI controller with a low-pass filter presented in Equation (17) can meet the velocity closed-loop control. After the rebuilding by the velocity loop, the characteristics will be further better, and we design the position controller to be an inertial element shown as Equation (18).

(17) $$C_{v}=\frac{K_{p}s+K_{i}}{s}\cdot \frac{1}{1+T_{f}s}$$

(18) $$C_{p}=\frac{K}{1+Ts}$$

## 5. Experimental Verification

Table 1 and Table 2 respectively descript parameters of the linear MEMS accelerometers and the CCD. Two linear MEMS accelerometers work as a group to get the angle acceleration of one direction in a differential configuration [12].

**Figure 7:**
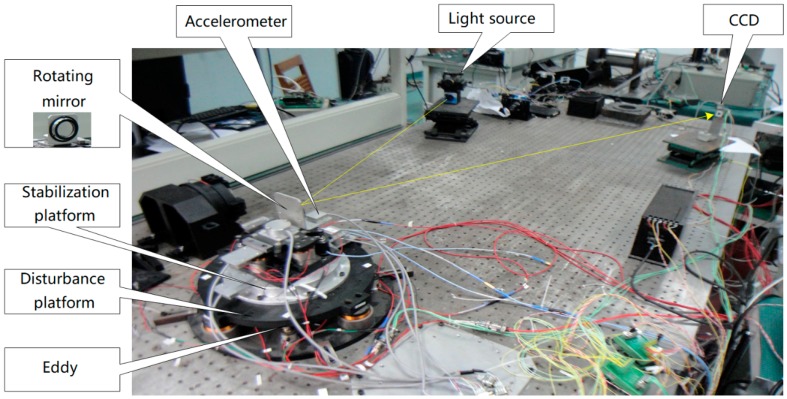
Experimental devices.

Figure 7 shows the experimental devices. The FTOS is a two-axis system. Due to the symmetry of the two axes, we focuses on one axis. To verify the above analysis, we use two FTOS platforms driven by voice coil motors. The upper stabilization platform to stabilize the LOS is mounted on the below disturbance platform which is utilized to simulate the outer disturbance. The outer disturbance is a sine signal produced by the dynamic signal analyzer. The light source emits light as a reference of LOS and the CCD receives the reflected light to provides the last LOS error. The MEMS accelerometers or FOG (XW-FG70-20, Beijing StarNeto Technology Co Ltd, Starneto, Beijing, China) are mounted on the stabilization platform to detect its angular acceleration or velocity, while the eddy is installed on the below platform to measure the given disturbance angle. All of the inertial sensors and the eddy have a sampling rate of 5000 Hz and the CCD updates in 100 Hz with 20 ms (2 frames) time delay. To get the DSA of the system, the stabilization platform should work on closed-loop mode and the disturbance platform works on open-loop mode. 

**Figure 8:**
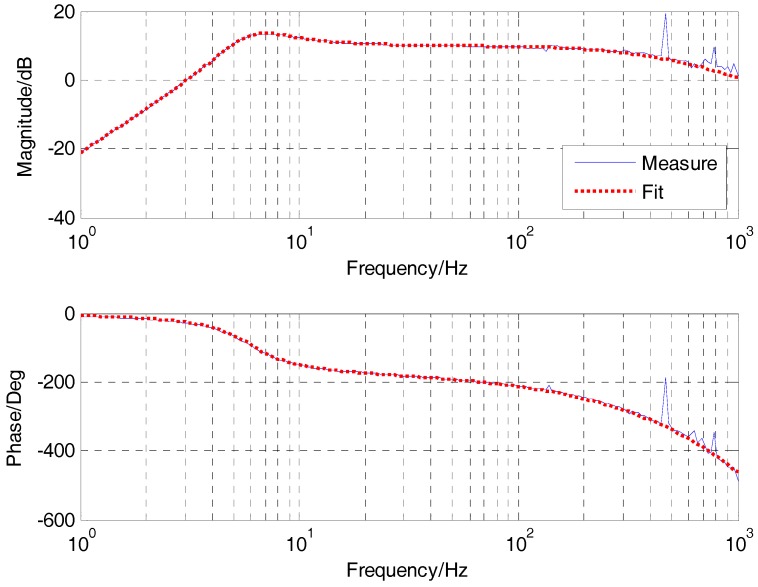
The acceleration open-loop bode response.

Figure 8 gives the acceleration open-loop bode response with the accelerometers, in which the fitting curve highly matches the actual. The acceleration open-loop transfer function is presented as follows:

(19) $${\tilde{G}}_{a}=\frac{0.0022s^{2}}{0.0007s^{2}+0.0185s+1}\cdot \frac{1}{0.0004s+1}$$

As in the previous analysis, we design the acceleration controller to be as Equation (20):

(20) $$C_{a}=150\cdot \frac{0.0007s^{2}+0.0185s+1}{s(1+0.00077s)}$$

In keeping with Equation (9), the actually used DOB controller is presented as Equation (21).

(21) $$C_{f}=\frac{0.00077s+1}{0.33s}$$

The velocity and position controller are descripted as follows:

(22) $$C_{v}=0.05\cdot \frac{0.33s+1}{s(1+0.001s)}$$

(23) $$C_{p}=0.012\cdot \frac{1}{1+0.0078s}$$

**Figure 9:**
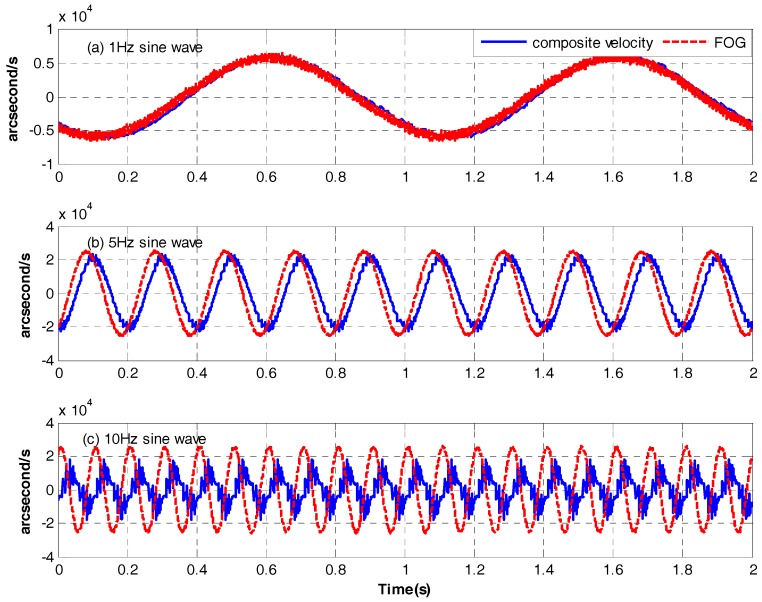
The time-domain curves of the composite velocity and FOG: (**a**) 1 Hz sine wave; (**b**) 5 Hz sine wave; (**c**) 10 Hz sine wave.

Figure 9 shows the time-domain curves of the composite velocity and FOG at different frequencies. Below 5 Hz, the phase lag brought by CCD’s time delay is too small to effect the DSA of FTOS. AS the frequency gose up, the negative effect will become bigger. However, according to Section 2.3, since the DOB method has greatly reformed the platform, the impact can be ignored.

**Figure 10:**
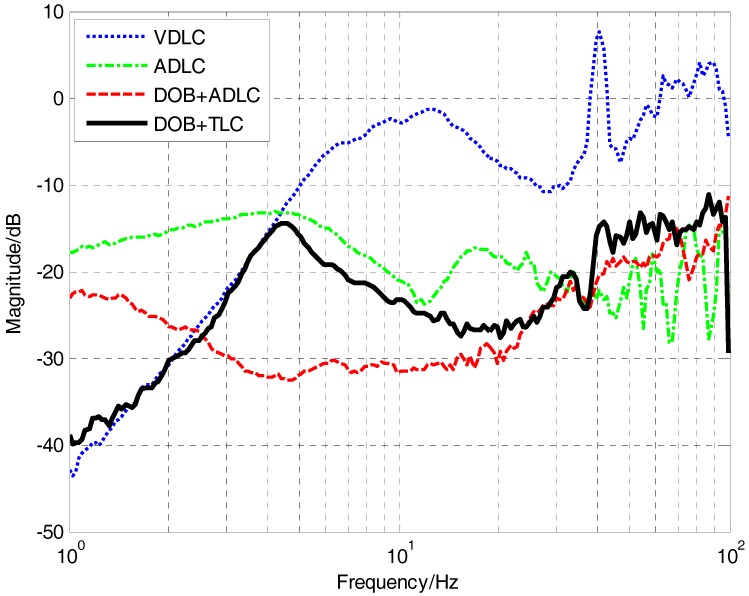
Disturbance attenuation of the four methods.

The total disturbance attenuation performance of the four methods are presented in Figure 10. The green line represents the pure ADLC method, whose DSA in low and medium frequency is not satisfied. Compared with the pure ADLC method, the only introduction of DOB could enhance the DSA in low and medium frequency, while the improvement in low frequency is a little, which is coincident with the previous analysis. After the composite velocity loop added to the system, the DSA below 2 Hz has increased a lot, which is very lacking in accelerometers. Although the time delay brought by the correction of the CCD would partly decrease the DSA in medium frequency, the last DSA in medium frequency still can be acceptable. Compared with the traditional VDLC method with FOG, the proposed TLC with DOB is obviously better in medium and high frequency. What’s more, in low frequency, they are close to each other. In general, with the introduction of the DOB and the composited velocity, the MEMS accelerometers could completely substitute the FOG and have a better comprehensive performance.

## 6. Conclusions

The main contribution of this paper is the substitution of sensors, whereby high-bandwidth, small-sized and cost-efficient MEMS accelerometers are used to replace traditional fiber-optic gyroscopes. The proposed method combines the DOB and the TLC only based on MEMS accelerometers and CCD to improve the DSA in FTOS. The DOB method is mainly used to enhance the DSA in medium frequency, and the composited velocity loop is to exchange parts of medium-frequency performance to low-frequency DSA, which can be applied in other occassions. What’s more, we combine the feedback control, DOB disturbance feedforward control and sensor fusion to fulfil the sensor’s potential. This fusion idea can also be transplanted to other sensors in further studies. Experiments verify that the proposed method has the best comprehensive performance and the MEMS accelerometers could totally substitute the FOG’s role in FTOS.

As the proposed method to correct the drift of the velocity integrated by accelerometers would bring the CCD’s delay to the velocity loop, the DSA in medium frequency is not perfect. Our future work will focus on further improving the medium-frequency performance. It will be a meaningful task to correct the drift and cut down the time delay influence of CCD.

## Figures and Tables

**Table 1:** MEMS accelerometers parameters.

Brand	Silicon Designs Inc.*
Model	Model 1221
Sensitivity	400 mV/g
Input Range	±10 g
RMS	10 $\mu g/\sqrt{Hz}$

* Kirkland, WA, USA.

**Table 2:** CCD parameters.

Brand	Pulnix *
Model	TMC-6740CL
Pixels	640 × 480
Pixel Size	7.4 μm

* National Instruments Corporation, Sunnyvale, CA, USA.
